# Antidiabetic Effects of Arginyl-Fructosyl-Glucose, a Nonsaponin Fraction from Ginseng Processing in Streptozotocin-Induced Type 2 Diabetic Mice through Regulating the PI3K/AKT/GSK-3*β* and Bcl-2/Bax Signaling Pathways

**DOI:** 10.1155/2020/3707904

**Published:** 2020-07-02

**Authors:** Xinglong Liu, Wencong Liu, Chuanbo Ding, Yingchun Zhao, Xueyan Chen, Sadia Khatoon, Yinan Zheng, Zhiqiang Cheng, Guangsheng Xi

**Affiliations:** ^1^College of Chinese Medicinal Materials, Jilin Agricultural University, Changchun 130118, China; ^2^College of Resources and Environment, Jilin Agricultural University, Changchun 130118, China; ^3^Jilin Agricultural Science and Technology University, Jilin 132101, China

## Abstract

Streptozotocin- (STZ-) induced type 2 diabetes mellitus (T2DM) caused insulin secretion disorder and hyperglycemia, further causing tissue and organ damage. In recent years, studies on ginseng (*Panax ginseng* C. A. Meyer) and its saponins (Ginsenosides) have proved to possess antidiabetic pharmacological activities, but the mechanism of nonsaponins on STZ-induced T2DM is still unclear. Arginyl-fructosyl-glucose (AFG) is a representative nonsaponin component produced in the processing of red ginseng. The present study was designed to assess the possible healing consequence of AFG on STZ-induced T2DM in mice and also to explore its fundamental molecular contrivances. T2DM-related indexes, fasting blood glucose levels, and body weight, histological changes, biochemical considerations, biomarkers, the mRNA countenance intensities of inflammatory facts, and variations in correlated protein manifestation in adipose tissue and liver tissue were calculated. Consequences specified that AFG usage successfully amends STZ-induced insulin conflict and liver grievance in T2DM. Systematically, AFG action diminished STZ-induced oxidative stress and inflammatory responses in the liver. In addition, we demonstrated that AFG also attenuates apoptosis and insulin secretion disorders in T2DM by adjusting the PI3K/AKT/GSK3*β* signaling pathway. At the end, these discoveries recommend that AFG averts the development of T2DM through numerous types of machinery and proposes that AFG can also be used in order to treat T2DM in the future.

## 1. Introduction

Diabetes mellitus (DM) is one of the most predominant health risk diseases, with incidence increasing rapidly every year [1], which has become a major public health problem in the world [2]. Type 2 diabetes mellitus (T2DM) is an important endocrine and metabolic disorder, which is caused by the impairment of insulin's ability to reduce blood glucose level due to the deficiency of insulin release and action, leading to hyperglycemia [3, 4], which is also the main clinical feature of T2DM [5, 6]. In addition, T2DM is caused by insulin resistance (IR) and pancreatic *β*-cell dysfunction, and IR is characterized by impaired insulin-mediated metabolism, which may also lead to many complications of inflammation and multiple organ damage [7]. There is no doubt that among all types of diabetes, T2DM poses a major threat to public health, accounting for 90% of all patients [8].

Currently, the mechanism of T2DM was extensively examined. Some studies have shown that insulin signaling pathway PI3K/AKT/GSK3*β* regulates blood glucose and participates in glucose metabolism in vivo. Drugs could improve T2DM by regulating multiple targets of the PI3K/Akt signaling pathway [9], enhancing the body's response to insulin. Consequently, one of the important healing targets for handling T2DM is PI3K/AKT/GSK3*β* signaling pathway. Furthermore, the INS/IGF-1 signaling pathway shows the most significant part in the evolution of insulin resistance (IR) by modifying the glucose breakdown of peripheral tissue, yet the Inhibition of GSK-3*β* in the liver is the main target to promote the utilization of glucose and improve insulin resistance. In fact, obesity increases the regularity of T2DM [10]. Obese DM could lead to dyslipidemia and abnormal expression of many genes related to lipid metabolism. At the same time, research has found that more and more patients with T2DM show cognitive decline or even dementia [11]. Genes that are involved in the development of T2DM symptoms may also lead to the development of Alzheimer's disease (AD) symptoms. It is of utmost importance to explain that the pathogenesis of T2DM is relatively complex. The patient's ability to produce insulin is not completely lost, but the role of insulin is poor, and effective drug intervention may contribute to keep this in balance, finally improving T2DM.

**Figure 1:**
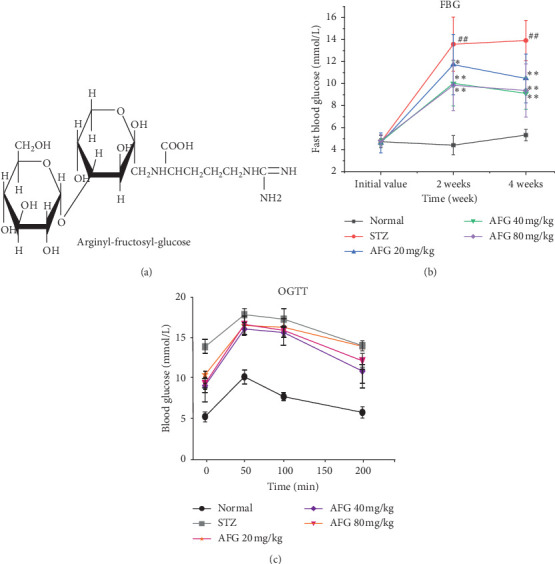
AFG improves the blood glucose index of T2DM. (a) the Structure of Arginyl-fructosyl-glucose (AFG); (b) Fasting blood glucose (FBG) levels in different doses mice at different times; (c) Trend line of oral glucose tolerance test (OGTT) in diabetic mice (DM). Data are exhibitedas the mean ± S.D. (*n* = 12). ^#^*P* < 0.05, ^##^*P* < 0.01, as equated with normal group; ^*∗*^*P* < 0.05, ^*∗∗*^*P* < 0.01, as equated with STZ group.

Actually, numerous research results indicate that the active ingredients in natural medicinal plants do well in improving T2DM [12]. Red ginseng (*Panax ginseng* Meyer) is evaporated from fresh ginseng by Maillard reaction and show high efficiency of inhibiting tumor growth, lowering blood glucose levels, antioxidant activity, and other pharmacological effects [13, 14], mainly caused by ginsenosides in red ginseng [15, 16]. In addition, some nonsaponins active substances are still produced during the processing, but there are fewer studies on these nonsaponins. Arginyl-fructosyl-glucose (AFG, Figure 1(a)) is an important nonsaponin active substance belonging to arginine derivatives [17–19], with good antioxidant activity [20], anti-inflammatory effects [21], etc. At the same time, research indicates that AFG has a strong protective effect on kidney injury [22]. Furthermore, it was reported to have hypoglycemic activity [23], but only the level of carbohydrate metabolizing enzymes in the serum has been initially studied, while the mechanism of its antihyperglycemia is still unclear. As a consequence, the purpose of this study is to focus on AFG as an important nonsaponin substance based on good antioxidation and anti-inflammatory effects was used to investigate the molecular mechanism of STZ-induced T2DM hypoglycemic effect.

## 2. Materials and Methods

### 2.1. Chemicals and Reagents

AFG was synthesized in the laboratory using the method of Li et al. [22], and the purity was 95.0% by the HPLC method. Commercial assay kits for aspartate transaminase (AST) and alanine aminotransferase (ALT), triglyceride (TG), total cholesterol (TC), malondialdehyde (MDA), glutathione disulfide (GSSG), superoxide dismutase (SOD), reduced glutathione (GSH) assay kit, low-density lipoprotein cholesterol (LDL-C), high-density lipoprotein cholesterol (HDL-C), protein extraction kits, and Hematoxylin-eosinstaining (HE staining) were got from Nanjing Jiancheng Bioengineering Co., Ltd (Nanjing, China). Masson staining kit and transferase UTP Nick end labeling (TUNEL) apoptosis detection kit were purchased from Beijing Solarbio Technology Co., Ltd. (Beijing, China). Streptozotocin (STZ) was obtained from Sigma, USA. Total RNA Rapid Extraction Kit, BioTeke Super RT Kit, 2x Power Taq PCR Master Mix, 2 × SYBP Real-Time PCR (RT-PCR) Premix was purchased from Beijing BioTeke Biotechnology Co., Ltd.

The antibody of rabbit monoclonal anti-mouse inducible nitric oxide synthase (iNOS), cyclooxygenase-2 (COX-2), and HRP-conjugated anti-mouse IgG, phosphatidylinositol 3-kinase (PI3K), p-PI3K, Protein kinase B (Akt), p-Akt, Glycogen synthase kinase-3*β* (GSK-3*β*), p-GSK-3*β*, b-associated X (Bax), b-cell-lymphoma-2 (Bcl-2), caspase-3, cleaved caspase-3, *β*-actin, and secondary antibodies for western blot were all obtained from Abcam (Cambridge, UK). Other compounds, such as alcohol of different investigative ranks were from Beijing Chemical Factory.

### 2.2. Animals and Experimental Protocol

70 male ICR mice, with weight ranges between 20–22 g, were given by Jilin Province Yisi laboratory animal technology limited company with License of Value no. of SCXK-(JI)-2016-0004 (Changchun, China). Ad libitum provided rodent laboratory food and tap water. These are kept under measured conditions with the light/dark cycle for 12 h at 25 ± 2°C and 60% ± 10% humidity, and all values were adjusted before one week of its usage. All the actions and experiments were performed on laboratory animals according to China law. All investigational processes were permitted for animals of the research laboratory of Jilin Agricultural University by a moral board on 15 June 2013 (Approval no. JLAU-ECLA-20130615).

In the research, normal group (*n* = 12) was nourished with common forage. Other mice were served with research diets (58% fat, 25.6% carbohydrate and 16.4% protein) for one month. They were continued to be given intraperitoneal injection of 100 mg/kg STZ citrate buffered saline solution (0.1 mol/L, pH 4.5) for a week. On the morning of the 7th day, their fasting blood glucose was determined. If ≥11.1 mmol/L, the model was successfully established [24]. The 48 DM mice with successful modeling were randomly divided into 4 groups with 12 mice in each group: STZ group (100 mg/kg) and AFG groups (80 mg/kg, 40 mg/kg, 20 mg/kg). The normal and STZ group mice were intragastrically administered 0.9% physiological saline at a dose of 0.01 mL/g. AFG dissolved in physiological saline was fed 20, 40, and 80 mg/kg by oral gavage once a day for 4 weeks. Body weight was determined every three days and fasting serum glucose was determined every 7 days [25].

At the end of the 4th week, all mice were sacrificed through cervical spondylolisthesis and rapidly dissected. Serum was separated by centrifugation (3500 rpm, 10 mins). The liver and spleen were collected to examine the appearance, size, and weight of organs. A minor part of tissue had been cut from the left portion of the liver of each mouse and preserved into 10% buffer formalin solution (m/v) for histopathological exam and liver tissues which remain were preserved at −80°C for index detection. Furthermore, abdominal adipose tissue of the mice was collected to detect other factors.

### 2.3. Fasting Blood Glucose and Oral Glucose Tolerance Test Levels

Fasting blood glucose (FBG) levels were measured on the day before dosing, followed by water intake in the evening and fasting on the 7th day. On the 8th morning, blood samples were collected by tail-vein sampling, examining FBG using blood glucose meter from Sinocare Biosensor Co., Ltd. (Changsha, China) [26]. Furthermore, the oral glucose tolerance test level was determined by Insulin Assay Kit (Nanjing Jiancheng Bioengineering Institute, Nanjing, China).

### 2.4. Biochemical Analysis

The serum AST and ALT, liver MDA, SOD, GSH, TC, TG, LDL-C, and HDL-C levels were determined by using commercially accessible indicative kits, according to Nanjing Jiancheng Institute of Biotechnology (Nanjing, China). Whole processes were done according to the companies manufacturing protocols.

### 2.5. Histopathological Examination and Immunohistochemical Analysis

After 4 weeks of treatment, the mice were sacrificed, and the liver of the mice was carefully removed and kept fixed in 4% formaldehyde solution. After fixation, ethanol was dehydrated, xylene was translucent and the liver was embedded in paraffin (make paraffin sections of about 3 *μ*m). After H&E staining, we placed this under a light microscope for observation for detecting the changes in histopathological analysis following the established protocol [27]. Masson staining was used to observe the interstitial fibers of liver tissue, which can focus on the level of interstitial fiber damage and interstitial lesions. For the assessment extent of fibrosis damage in the livers, sections were stained with Masson staining. The fibrotic area was assessed using a light microscope and the Quantity One computerized morphometry system (Bio-Rad, Hercules, USA). To observe immunohistological staining of the liver, the liver samples sealed in paraffin were cut to a thickness of 5 *μ*m, and then the sections were permeabilized for antigen retrieval. Subsequently, the slides were incubated for 1.5 h under 1% BSA and further incubated with primary antibodies to iNOS (1 : 200) or COX-2 (1 : 200) at 4°C for 12 h. Subsequently, the sections were washed with PBS and then incubated with secondary antibody (Abcam, Cambridge, UK). After rinsing, the Elivison two-step method was performed for immunohistochemical staining. An optical microscope was used to observe the slices (200×).

### 2.6. TUNEL Staining Analysis

To accurately reflect the morphological characteristics of apoptotic cells and apoptotic bodies in situ, TUNEL staining had been widely used in the study of apoptotic cells. In this research, apoptotic cells in liver tissue were measured by TUNEL staining kit, and the apoptotic cells were calculated by microscopy.

### 2.7. Quantitative Real-Time PCR Analysis

The analysis of the genes expression in the liver and abdominal adipose tissue is done by RT-PCR. After the mice were sacrificed, the liver and adipose tissue were, respectively, wrapped in foil paper and labeled. Samples were preserved in the refrigerator at −80°C. For RNA extraction a part of the liver tissue was taken, crushed into powder in liquid nitrogen, and then transferred to the 1.5 mL centrifuge tube. Then 1 mL lysate was added and shaken. Total sample RNA was collected according to the high purity total RNA Rapid Extraction Kit instructions, RNA concentration was measured, and the total RNA extracted was subjected to cDNA synthesis according to Bio Take RT Kit manual. The fluorescence quantitative primer sequences are shown in Table 1. According to the primer synthesis instructions, the primers were diluted and used as the reaction solution (diluted 1-fold with 10-fold dilution), according to 2 × Power Taq PCR Master Mixshi kit PCR amplification, according to 2 × SYBP real-time PCR premixture kit manual quantitative analysis of quantitative RT-PCR.

### 2.8. Western Blotting

The mice livers were regimented in radio-immunoprecipitation assay buffer at 4°C and supernatant was taken after the accomplishment of centrifugation (21000 × *g*) for 30 min at 4°C. The quantity of total protein was enumerated by using a nucleic acid protein determination system (Thermo Fisher, USA). Samples were further dealt with on SDS-PAGE (loaded 30 *μ*g protein extract) and then transported to polyvinylidene fluoride (PVDF) membranes (65421, MilliporeInc, USA) [28]. Membranes were blocked with 5% nonfat dry milk in 1 × TBS (0.1% Tween-20) for 30 min and then incubated overnight with the primary antibodies at 4°C, including Bcl-2, Bax, cleaved caspase-3, caspase-3, PI3K, P-PI3K, AKT, P-AKT, GSK-3*β*, and P-GSK-3*β*. After incubation with horseradish peroxidase-labeled secondary antibody for 60 min, signals were then identified with a heightened chemiluminescence system (Amersham). At the end, after incubation with horseradish peroxidase-labeled secondary antibody for 60 min, by using a Gel Imaging analyzer (Gel Doc XR, BIO-RAD Inc., USA), the density of each band was determined [29].

### 2.9. Statistical Analysis

With three self-governing repetitions, all experiments were consummated. The figures were designed by GraphPad Prism 7.0 (ISI, USA) and Adobe illustrator cc2017 (ADOBE, USA). Data were expressed as mean ± standard deviation (SD) and the statistical implication was scrutinized through a one-way examination of variance (ANOVA) valuation using SPSS 17.0 software (SPSS Inc., Chicago, IL, USA). *P* < 0.05 was considered as significant or *P* < 0.01 as very significant.

## 3. Results

### 3.1. Effects of AFG on Body Weight and Liver Index in Mice

During the course of this research, as shown in Table 2, before STZ induction, the body weight of all groups in mice was relatively close, almost no difference. After STZ injection and the four weeks of administration, the mice in the normal group gained 1.60 g. Yet, compared with the normal group, the body weight of the STZ group decreased sharply (*P* < 0.01), which was 3.26 g lower than the initial body weight, indicating that STZ seriously affected the growth and development of the mouse body. At the same time, the mice in each dose group of AFG had a steady increase in body weight. In addition, it is not difficult to find that with the increase of the dose of AFG, the body weight of the mice in the treatment group also tends to increase, and the weight of the AFG 80 mg group increased the most, reaching 0.70 g. Compared with the normal group, the body weight of the model group by STZ induction was significantly reduced (*P* < 0.05). The results indicate that AFG treatment can reverse the weight loss of STZ-induced diabetic mice in a dose-dependent manner.

Correspondingly, the liver index and spleen index of diabetic mice also vary to varying degrees. After STZ induction, the liver index and spleen index of STZ group reached 63.90 mg/g and 5.37 mg/g, respectively, which were significantly increased compared with the blank group (*P* < 0.05). The liver index and spleen index of the mice in each dose group of AFG decreased slightly, and the effect of AFG 80 mg/kg was significantly higher than that of the STZ group (*P* < 0.05). In summary, these data indicate that AFG treatment can improve signs and organ tissue changes of STZ-induced diabetic mice.

### 3.2. Effects of AFG on Fast Blood Glucose and Oral Glucose Tolerance Test Levels

To evaluate the tupgrading of AFG in STZ-induced DM, the changes in fasting blood glucose (FBG) were measured at different times after administration. As shown in Figure 1(b), two weeks after the injection of STZ, there was no significant change in the FBG levels in the normal group, whereas FBG levels in the STZ group increased sharply compared with the normal group (*P* < 0.05). The FBG levels in the AFG groups also increased, but compared with the model group, AFG treatment had a significant decrease (*P* < 0.05), and the AFG 80 mg/kg dose group had the lowest FBG level, which was 30% lower than the STZ group. After 4 weeks of AFG treatment, the level of FBG in the STZ group continued to increase, while AFG group was significantly lower than that in the STZ group (*P* < 0.05). The results indicated that STZ-induced changes in diabetic blood glucose levels could be effectively regulated after AFG treatment.

The trend line of oral glucose tolerance test (OGTT) of AFG to diabetic mice is shown in Figure 1(c). After half an hour of the oral administration of glucose, the blood glucose level of each group of mice shows an increasing value. After 1 h, the blood glucose level of mice in each group began to decline gradually until 2 h and dropped back to the original value, and we found that the effect of OGTT in the middle dose of AFG was the most noticeable. Therefrom, we also found positive effects of AFG administration in STZ-induced DM.

### 3.3. AFG Attenuates STZ-Induced Diabetic Liver Injury and Histopathological Examination

**Figure 2:**
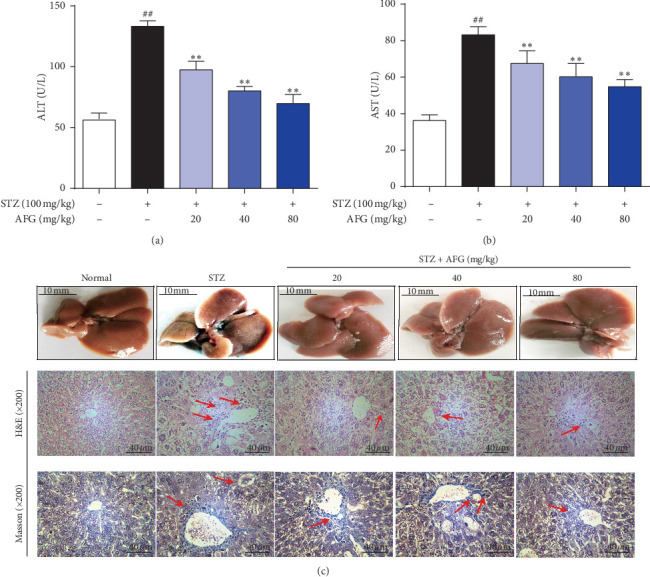
Protective effect of AFG treatment on STZ-induced liver injury in T2DM mice. (a) Alanine aminotransferase (ALT) content in mice liver; (b) Aspartate aminotransferase (AST) content; (c) H&E and Masson staining of liver sections, magnification: ×400. Data explored as the mean ± S.D. (*n* = 12). ^#^*P* < 0.05, ^##^*P* < 0.01, as equated with normal group; ^*∗*^*P* < 0.05, ^*∗∗*^*P* < 0.01, as equated with STZ.

To evaluate the protective effect of AFG on STZ-induced diabetic liver injury in mice, serum levels of ALT and AST were measured. STZ-induced heights of ALT and AST in the model group considerably amplified as equated to the normal group (Figures 2(a) and 2(b)). As shown in Figure 2(c), H&E showed representative micrographs of the liver obtained from different treatment groups. Typical pathological features, including necrosis, inflammatory infiltration, and extensive vacuolar degeneration, were found in the STZ group, suggesting liver damage caused by diabetes mellitus. The results showed that the hepatic necrosis and inflammatory cell infiltration decreased in the treatment groups of 40 mg/kg and 80 mg/kg AFG, suggesting that AFG pretreatment plays a protective role in STZ-induced cell injury. In Masson staining, a large number of fat vacuoles were found in the hepatic tissues of the STZ group, and the extracellular matrix components were excessively deposited. The lipid droplets of the hepatocytes and mitochondrial damage were significantly decreased with AFG treatment, and the endoplasmic reticulum was increased significantly and orderly arrangement. Therefore, we speculated that AFG pretreatment may improve liver injury induced by T2DM.

### 3.4. AFG Alleviates STZ-Induced Oxidative Stress and Lipid Metabolism Disorders

**Figure 3:**
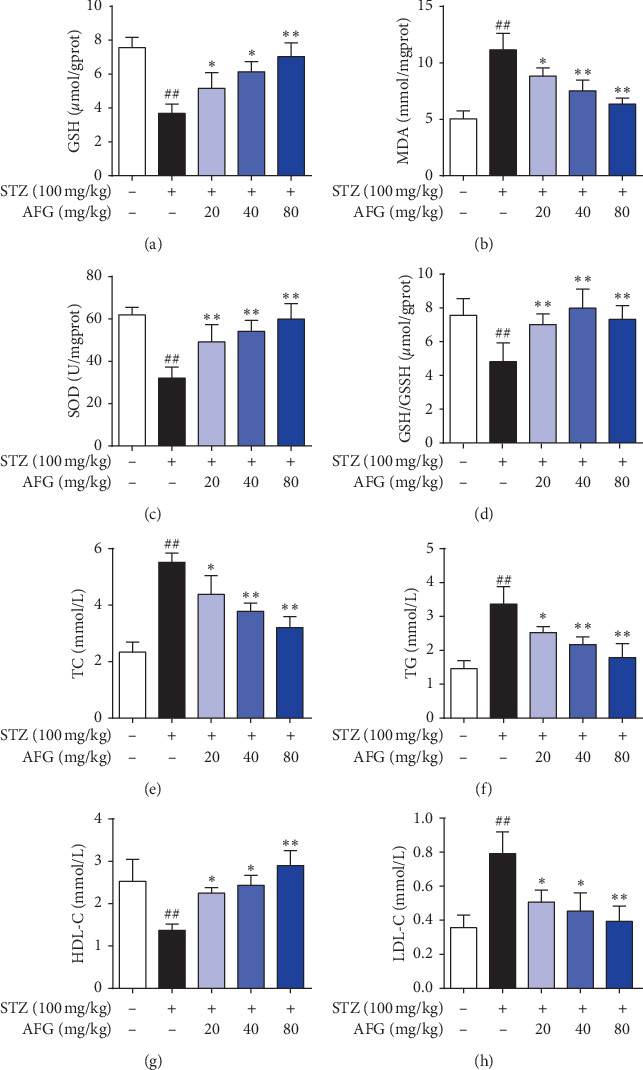
AFG mitigates oxidative stress and lipid metabolism disorder in STZ-induced T2DM. (a) Liver GSH activities; (b) Lipid peroxidation MDA; (c) Antioxidant enzyme SOD; (d) Liver GSH/GSSH level; (e–h) Effect of AFG on the expression of TC, TG, LDL-C and HDL-C in liver. Data presented as the mean ± S.D. (*n* = 12). ^#^*P* < 0.05, ^##^*P* < 0.01, as equated with normal group; ^*∗*^*P* < 0.05, ^*∗∗*^*P* < 0.01, as equated with STZ group.

Diabetes and its impediments cause oxidative stress damage and lipid metabolism disorders. Quantities of MDA, SOD, GSH, and GSSH level in order to define the consequence of AFG on oxidative stress in STZ-induced DM. MDA was frequently used as a biomarker for liver oxidative harm and also one of the lipid peroxidation yields. When compared with the normal group, MDA was meaningfully increased by 63.5% in the STZ group (*P* < 0.01), but AFG usage ominously repressed MDA in the liver compared with the STZ group (*P* < 0.01, Figure 3(b)), and the AFG 80 mg/kg dose group was the most effective, which was 43.8% lower than the STZ group. Meanwhile, in STZ-treated liver, the activity of SOD and GSH was decreased, while they were significantly increased by AFG treatment (*P* < 0.05, Figures 3(a), 3(c), and 3(d)). These data suggested that AFG attenuated STZ-induced oxidative stress damage in DM mice. Furthermore, TG, TC, LDL-C, and HDL-C were biochemical indicators related to tissue lipid metabolism, demonstrating the level of lipid peroxidation. In the STZ group, TG, TC, and LDL-C levels were suggestively increased and HDL-C was meaningfully debased by 44.2% compared with the normal group (*P* < 0.01, Figures 3(e)–3(h)). However, compared with the STZ, TG, TC, and LDL-C levels were significantly suppressed, and the HDL-C had a different degree of increase after AFG treatment (*P* < 0.05), while the AFG 80 mg/kg had a very significant increase (*P* < 0.01). In conclusion, these pieces of evidence indicated that AFG attenuated oxidative stress damage and lipid metabolism disorders in STZ-induced diabetic mice.

### 3.5. AFG Attenuates STZ-Induced Inflammation in the Liver

**Figure 4:**
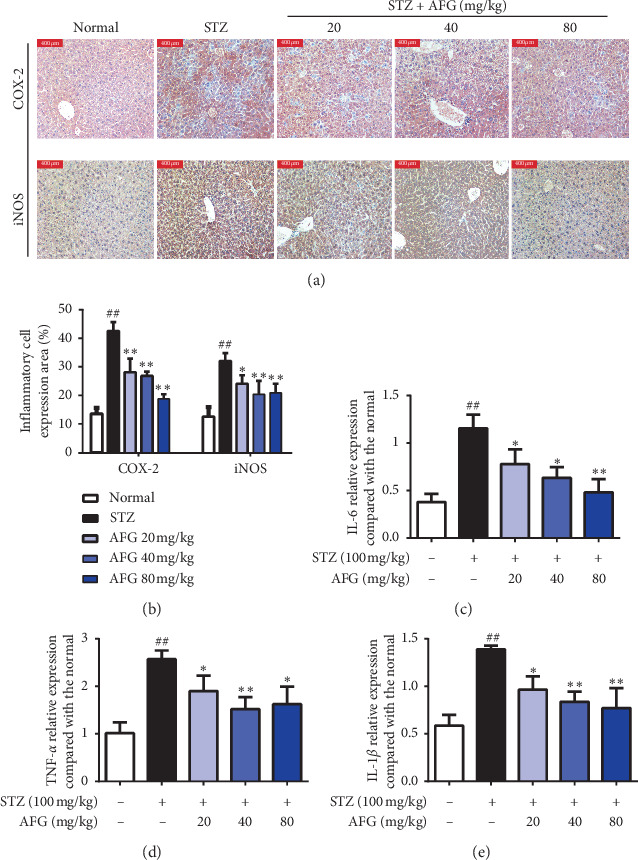
Effects of AFG on the expression of inflammatory factors in liver tissue. (a, b) Analysis of iNOS and COX-2 expression in liver tissue. (c–e) Relative mRNA levels of TNF-*α*, IL-1*β,* and IL-6 in liver tissue. Data presented as the mean ± S.D. (*n* = 12). ^#^*P* < 0.05, ^##^*P* < 0.01, as equated with the normal group; ^*∗*^*P* < 0.05, ^*∗∗*^*P* < 0.01, as equated with the STZ group.

Inflammatory factors were involved in STZ-induced tissue damage in diabetic mice. In addition, other studies have shown that oxidative stress damage was associated with the production of proinflammatory factors [30]. In this research, we detected the expression levels of TNF-*α*, IL-1*β,* and IL-6 proinflammatory factors in liver tissues by rt-PCR analysis. The results showed that STZ-induced diabetic mice had a significant increased levels of TNF-*α*, IL-1*β* and IL-6 in the liver (*P* < 0.01, Figures 4(c)–4(e)), whereas treatment with AFG significantly inhibited the overexpression of TNF-*α*, IL-1*β,* and IL-6 (*P* < 0.01).

Importantly, to further understand the anti-inflammatory effects of AFG, immunohistochemical staining was used to analyze the expression of inflammatory cytokines in liver tissues. As shown in Figures 4(a) and 4(b), we observed that it was significantly obvious that the positive staining of iNOS and COX-2 in liver cytoplasm of STZ-induced diabetic mice compared with the normal group. However, the expression of iNOS and COX-2 was significantly inhibited in the liver of diabetic mice administration of AFG treatment with 20, 40, and 80 mg/kg and dosing dose dependence. These results demonstrated that the protective effect of AFG on STZ-induced diabetic mice may be related to its anti-inflammatory.

### 3.6. AFG Ameliorates STZ-Induced Apoptosis

**Figure 5:**
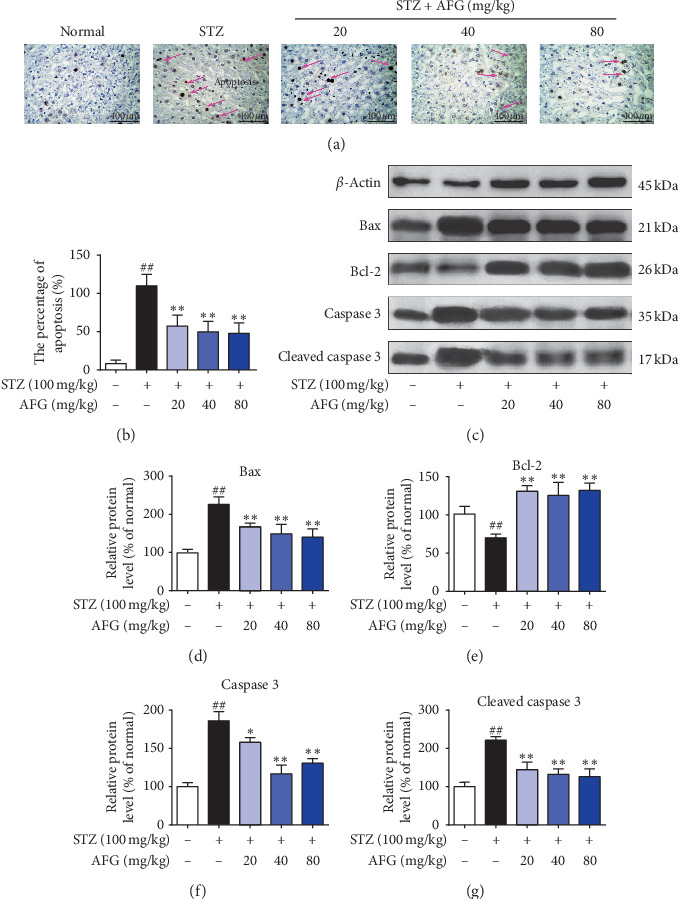
Effects of AFG antiapoptotic in STZ-induced liver tissue. (a, b) Evaluation of TUNEL staining and the percentage of apoptosis. (c) Determination of Bax, Bcl-2, caspase-3, Cleaved caspase-3 proteins expression, and *β*-actin as a reference. (d–g) Analysis of relative protein expression by integrated optical density. Data presented as the mean ± S.D. (*n* = 12). ^#^*P* < 0.05, ^##^*P* < 0.01, as equated with the normal group; ^*∗*^*P* < 0.05, ^*∗∗*^*P* < 0.01, as equated with the STZ group.

TUNEL staining could stain apoptotic nuclei or apoptotic bodies. The research was used to determine the apoptotic level of hepatocytes. As shown in Figures 5(a) and 5(b), almost no apoptotic cells were observed in the liver cells of the normal group, but compared with the normal group, the number of TUNEL-positive cells was significantly increased in the STZ-induced model group (*P* < 0.01). In contrast, treatment with AFG significantly reduced the expression of TUNEL-positive cells in hepatocytes. Furthermore, to further detect the effect of AFG on hepatocyte apoptosis, western blotting was used to determine the proapoptotic factor Bax and antiapoptotic factor Bcl-2 and the caspase-3 protein factor related to apoptosis. The results showed that the expression of Bax and Cleaved caspase-3 was significantly increased after STZ induction, but the expression of both proteins was reversed by AFG pretreatment. In addition, the positive expression of Bcl-2 was significantly inhibited after STZ treatment. Bcl-2 levels were significantly higher in the model group, which was contrary to the expression of the proapoptotic factor Bax, indicating that AFG prevented the effects of STZ-induced hepatocyte apoptosis in diabetic mice.

**Figure 6:**
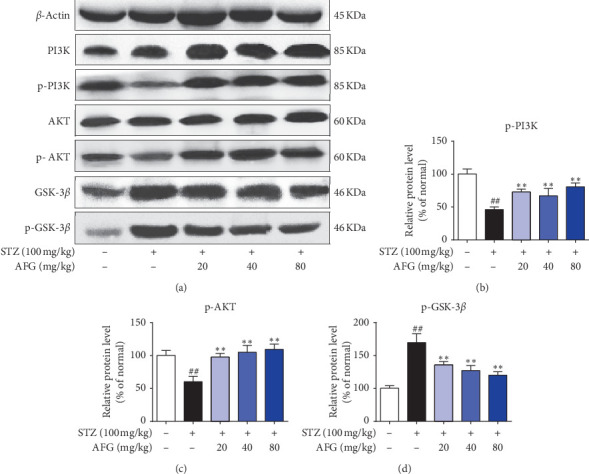
AFG regulates glycogen metabolism and ameliorates hepatocyte apoptosis in diabetic mice by adjusting the liver PI3K/AKT/GSK3*β* signaling pathway. (a) Possessions of AFG on the manifestation of PI3K/AKT/GSK3*β* in the liver tissues were explored by western blot exploration. (b–d) Densitometric examination of western blot. Data are expressed as the mean ± S.D. (*n* = 10). ^#^*P* < 0.05, ^##^*P* < 0.01, as equated with the normal group; ^*∗*^*P* < 0.05, ^*∗∗*^*P* < 0.01, as equated with the STZ group.

In addition, to further explore the molecular mechanism of AFG in improving STZ-induced T2DM, we observed PI3K/AKT/GSK3*β* signaling pathway. PI3K/AKT was an important signal transduction factor to improve apoptosis, and GSK3*β* as its downstream signal could not only promote cell survival but also regulate insulin signal transduction and body blood glucose level. As shown in Figures 6(a)–6(d), western blot results showed that the levels of P-PI3K and P-AKT decreased and P-GSK3*β* increased compared with the normal group (*P* < 0.01), but these changes were significantly reversed after AFG treatment (*P* < 0.01), indicating that AFG not only regulated insulin level but also inhibited apoptosis by regulating PI3K/AKT/GSK3*β* signaling pathway.

### 3.7. AFG Regulates STZ-Induced Changes Peritoneal Fat and Liver Factors in T2DM Mice

**Figure 7:**
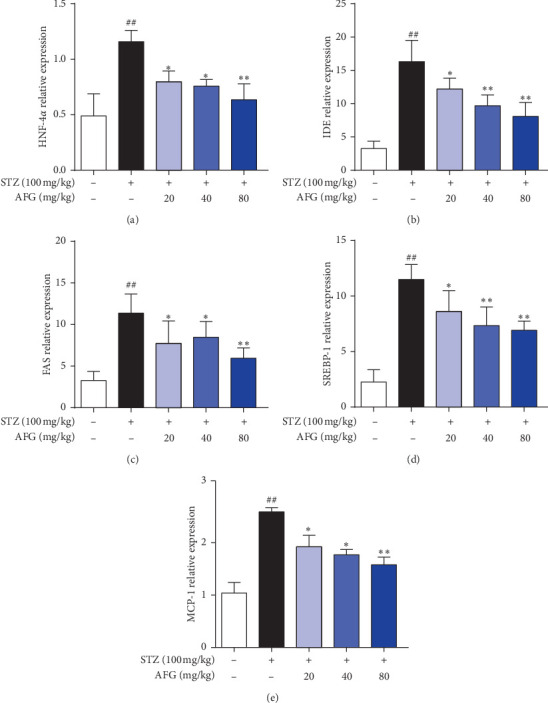
AFG improves STZ-induced changes in peritoneal fat and liver factors in T2DM mice. (a, c, e) Effects of AFG on the expression of SREBP-1, FAS, and MCP-1 in the peritoneal fat were measured by RT-PCR. (b, d) Special effects of AFG on the manifestation of IDE and HNF-4*α* in the liver tissue were measured by RT-PCR. Data are conveyed as the mean ± S.D. (*n* = 12). ^#^*P* < 0.05, ^##^*P* < 0.01, as equated with the normal group; ^*∗*^*P* < 0.05, ^*∗∗*^*P* < 0.01, as equated with the STZ group.

As revealed in Figures 7(a), 7(c), and 7(e), the effects of AFG on SREBP-1, FAS, and MCP-1 mRNA expression in mice peritoneal fat, the relative countenance level of the model group was ominously greater than other groups (*P* < 0.01). Among the SREBP-1 gene, in the STZ group when compared with the AFG 40 and 80 mg/kg groups, there was a significant difference (*P* < 0.01). In the FAS gene, AFG 20 mg/kg was significantly different from the model group (*P* < 0.05), while in the high was significantly different from the model group (*P* < 0.01). Compared with the STZ group, the relative expression levels of the AFG of three groups were significantly reduced (*P* < 0.05). Additionally, based on the effects of AFG on IDE and HNF-4*α* mRNA expression in T2DM mice liver tissue, it could be seen from Figures 7(b) and 7(d) that the IDE and HNF-4*α* relative genes expression of each treatment group was decreased to some level when equated to the STZ group, which clearly shows that AFG regulated IDE and HNF-4*α* and attenuated diabetes mellitus, and the difference was statistically significant.

## 4. Discussion

Numerous studies have demonstrated that diabetes is a complex metabolic disorder and a major problem that threatens human health [31]. At present, chemical drugs are used to treat diabetes, which produces various toxic and side effects. However, some active ingredients extracted from natural plants can not only improve diabetes and its complications but also be safer and have fewer side effects. As mentioned above, as an intermediate product in the process of red ginseng [17], AFG is also a representative nonsaponin active constituents in red ginseng, and it has been proven to have anti-inflammatory, antioxidative stress damage, and antihypertensive and other pharmacological effects. This study has confirmed that AFG has a good effect on STZ-induced T2DM inflammatory response, oxidative stress damage, and apoptosis.

In general, in the STZ-induced T2DM model, ALT and AST are released into the blood stream due to liver damage in hepatocytes, resulting in a significant increase in ALT and AST [32]. This research confirmed that serum levels of ALT and AST were significantly increased after STZ-induced, indicating early liver injury in T2DM, and raised serum levels of that enzyme indicated hepatobiliary disease [33]. In contrast, treatment with different doses of AFG significantly inhibited ALT and AST levels, which means that AFG improved liver disease in STZ-induced diabetic mice. Meanwhile, the histopathological examination confirmed the improvement of liver tissue abnormalities by AFG. Besides, our study also found that AFG treatment significantly ameliorated body weight change, glucose tolerance, and hyperglycemia in T2DM.

At the same time, in the liver and serum of diabetic patients due to the finding of lipid peroxidation markers, the participation of oxidative stress and lipid peroxidation in the pathogenesis of T2DM is confirmed. In diabetic patients, the degree of lipid peroxidation in the liver will increase, and liver cells will be damaged. In severe cases, liver steatosis, inflammation, hepatitis, and liver failure will happen [34]. On the initial stage of liver ailment, TC and TG accumulate in hepatocytes and cause the growth of fatty liver [35]. The results of this study showed that different doses of AFG could significantly inhibit the increase of TC and TG in DM mice, indicating that AFG improved the liver disease of STZ-induced diabetic mice. Furthermore, current research indicates that severe DM patients can cause hyperlipidemia and atherosclerosis. HDL-C is an antiatherosclerotic plasma lipoprotein that transports cholesterol from surrounding tissues, including the arterial wall, to the liver for metabolism. LDL-C is the primary atherogenic lipoprotein in plasma lipoprotein and easily penetrates the intima of the artery. It is easily oxidized and has a stronger atherogenic effect. SOD can remove superoxide anion from the body, enhance cell activity, and enhance immunity, while SOD catalyzes the elimination of superoxide radicals through continuous oxidation and reduction of metal ions of transition state [36]. MDA is widely used as a marker of lipid peroxidation and a major parameter for the status of oxidative stress [37]. It has been reported that in rodent models, MDA levels are increased in the occurrence of oxidative hassle in the liver [38]. In this paper, the results publicized that AFG increased the activity of SOD and GSH in DM mice, decreased the content of MDA and LDL-C, and increased HDL-C level, indicating that AFG protected STZ-induced DM mice by enhancing immunity, reducing lipid peroxidation, and eliminating free radicals.

In addition, another important pathogenesis of T2DM is inflammation. Inflammation is a defensive challenge by injured tissues to eliminate harmful stimuli and treat wounds. Meanwhile, inflammation is also a multifaceted occurrence relating to a variety of cellular and molecular interfaces that are firmly organized to prevent different pathologies and diseases [39]. The TNF-*α* gene is a chief element in many physiological and pathological changes and the gene affects the action and countenance of transporters by motivating invulnerable cell permeation and cell death, and it also facilitates tissue impairment [40]. Some researchers have shown that elevated levels of TNF-*α* gene directly affect the insulin receptor signaling, reduce insulin sensitivity, and reduce insulin receptors and insulin receptor substrate contact ultimately leading to insulin confrontation [41]. Therefore, we analyzed the expression of TNF-*α*, IL-6, and IL-1*β* inflammatory factors in the liver of DM mice by RT-PCR. The results showed that STZ-induced excessive expression of inflammatory factors in the liver of T2DM, while AFG treatment significantly inhibited the expression of inflammatory factors and formed a protective effect. Similar results were obtained by immunohistochemical analysis of iNOS and COX-2 proinflammatory factors.

Previous studies have shown that STZ-induced T2DM is closely related to liver cell necrosis and apoptosis. In this study, the expression of apoptosis-related proteins in the liver tissue of T2DM mice was quantified by western blot. PI3K is an intracellular phosphoinositol kinase, which participates in cell differentiation, proliferation, and apoptosis. As an important downstream signal of PI3K, AKT can respond to its function by regulating the BCL-2/bax and caspase signaling pathways. These protein markers are important factors regulating apoptosis. GSK-3*β* is an insulin signaling regulator that plays an important negative regulatory role in diabetic insulin signaling, and its enhanced expression exacerbates T2DM insulin resistance [42]. Other studies have shown that GSK-3*β*, as a downstream signaling factor of PI3K/AKT, not only regulates insulin signaling but also participates in apoptosis [43]. When PI3K/AKT is activated, it produces its phosphorylated substance, which activates downstream signaling factors, regulates GSK-3*β* and antiapoptotic factors, and inhibits apoptosis. Besides, STZ-induced can induce tissue damage by activating Bcl-2 and caspase-3 related factors and interact with inflammatory factors to regulate apoptosis. In the research, western blot exploration exposed that phosphorylation points of PI3K, AKT were seemingly withdrawn in the model group as compared to the normal group, and GSK-3*β* was increased. After AFG treatment, the P-PI3K, P-AKT levels were significantly increased, yet P-GSK3*β* was attenuated, apoptosis in liver tissue was reduced, and TUNEL staining confirmed the results. These results also indicated that the improving T2DM effect of AFG is dependent on the suppression of the PI3K/AKT/GSK3*β* signaling pathway.

At the same time, combined with previous studies, insulin resistance (IR) is a major feature of T2DM. Analysis of T2DM-related genes will help to further explore its pathogenesis. The IDE gene is a metalloproteinase present in cells that cleaves the insulin chain and inactivates it [44]. Studies have shown that as compared with normal people, the IDE activity of T2DM is significantly increased, indicating that elevated IDE activity may be one of the causes of insulin resistance [45], and IDE gene mutations will change the functional products expressed by IDE, abnormal degradation of insulin, ultimately leading to IR and high blood glucose. HNF-4*α* is highly expressed in liver tissues and involved in the regulation of glycolipid metabolism and insulin secretion, which may play an important role in diabetic liver injury [46]. As a transcription factor regulating gene-specific expression in the liver, hepatocyte nuclear factor can directly enter the nucleus to regulate the expression of the liver and pancreatic islet related genes. The results of this study indicated that AFG reduced the expression of IDE and HNF-4*α* genes in T2DM liver and improved the symptoms of T2DM insulin degradation caused by the increased expression of the IDE gene. SREBP-1 has been shown to be closely associated with IR [47]. Decreased insulin sensitivity in the liver leads to elevated liver glucose production, hyperinsulinemia, increased beta-cell mass, and hyperglycemia [48]. It has been reported that SREBP-1 is an important factor regulating lipid metabolism disorder and is tangled in the regulation of lipid breakdown of key enzymes such as FAS to catalyze the synthesis of long-chain fatty acids [49]. The involvement of Fas on cells leads to the formation of death-induced active protease complex (DISC), which cleaves caspase and induces apoptosis [50–52]. The FAS gene is increasingly expressed in peritoneal fat, the liver, and other tissues. It converts carbohydrates into fatty acids and stores them in TG form. In recent years, research has shown that monocyte chemotactic factor (MCP-1) plays an important role in promoting inflammation in diabetic adipose tissue. Under the action of chemokine MCP-1, the movement of macrophages can lead to the release of inflammatory factors, which initiates the inflammatory response, promotes the process of inflammation, and aggravates the damage of the body's function. The results of this study showed that AFG effectively reduced the expression of SREBP-1, FAS, and MCP-1 genes in DM mice, inhibited inflammatory reactions, reduced fat accumulation, and improved insulin resistance.

## 5. Conclusions

In conclusion, this study demonstrated that AFG regulates diabetic symptoms through multiple mechanisms in mice. Obviously, AFG improves diabetes by decreasing STZ-induced oxidative stress injury, inflammation, and cell apoptosis. Therefore, it is concluded that AFG prevents T2DM and IR by regulating the mechanism related to PI3K/AKT/GSK3*β* signaling transduction pathway. The consequences designate that AFG has the potential to improve the treatment of T2DM and may provide a new mechanism for the treatment of insulin resistance.

## Figures and Tables

**Table 1:** Fluorescence quantification primer sequence.

Gene	Primer sequencing (forward and reverse)	
TNF-*α*	Forward	5′-TGGCAAATGTGAGAAACGAG-3′
	Reverse	5′-AAACCAGAACAGACCCAACG-3′

IL-1*β*	Forward	5′-TCCAGGATGAGGACATGAGCAC-3′
	Reverse	5′-GAACGTCACACACCAGCAGGTTA-3′

IL-6	Forward	5′-CCACTTCACAAGTCGGAGGCTTA-3′
	Reverse	5′-CCAGTTTGGTAGCATCCATCATTTC-3′

IDE	Forward	5′-TCCCATACCAGACCTTCAGC-3′
	Reverse	5′-GTATTCACCCAGCCCTTTGA-3′

FAS	Forward	5′-ACTGCGATTCTTCTGGCTGT-3′
	Reverse	5′-GCGATTTCTGGGACTTTGTT-3′

SREBP-1	Forward	5′-AACCAGAAGCTCAAGCAGGA-3′
	Reverse	5′-TCATGCCCTCCATAGACACA-3′

Mcp-1	Forward	5′-CTGTGCTGACCCCAATAAGGA-3′
	Reverse	5′-ACAGAAGTGCTTGAGGTGGT-3′

HNF-4*α*	Forward	5′-AAATGTGCAGGTGTTGACCA-3′
	Reverse	5′-CACGCTCCTCCTGAAGAATC-3′

*β*-actin	Forward	5′-GTGCTATGTTGCTCTAGACTTCG-3′
	Reverse	5′-ATGCCACAGGATTCCATACC-3′

**Table 2:** Effects of AFG on body weight of DM mice ($\bar{x}\pm s$, *n* = 12).

Group	Dosage (mg/kg)	Before administration	After administration			
			4 weeks	Weight gain (g)	Liver index (mg/g)	Spleen index (mg/g)
Normal	—	38.75 ± 3.29	40.35 ± 2.92	1.60	47.10 ± 2.87	3.43 ± 0.90
STZ	—	38.94 ± 4.51	35.68 ± 2.49^##^	−3.26	63.90 ± 4.78^##^	5.37 ± 0.73^#^
AFG	20	38.91 ± 2.77	38.94 ± 2.86	0.03	50.21 ± 4.35^*∗∗*^	4.64 ± 0.84
	40	38.09 ± 3.63	38.31 ± 3.94	0.22	48.93 ± 3.94^*∗∗*^	4.41 ± 0.65
	80	38.81 ± 3.49	39.51 ± 3.18^*∗*^	0.70	50.74 ± 5.38^*∗∗*^	3.66 ± 0.57^*∗*^

^#^ *P* < 0.05, ^##^*P* < 0.01, as equated with the normal group; ^*∗*^*P* < 0.05, ^*∗∗*^*P* < 0.01, as equated with the STZ group. The same as follows.

## Data Availability

All data used in this study are provided in this article. All data are available from the corresponding author.
